# Editorial: Bioinformatics analysis of omics data for biomarker identification in clinical research, Volume II

**DOI:** 10.3389/fgene.2023.1256468

**Published:** 2023-07-24

**Authors:** Mengtao Sun, Lusheng Li, Hanyu Xiao, Junxi Feng, Jieqiong Wang, Shibiao Wan

**Affiliations:** ^1^ Department of Genetics, Cell Biology and Anatomy, University of Nebraska Medical Center, Omaha, NE, United States; ^2^ Department of Biostatistics, Harvard School of Public Health, Boston, MA, United States; ^3^ Department of Neurological Sciences, University of Nebraska Medical Center, Omaha, NE, United States

**Keywords:** omics data, biomarker, machine learning, clinical research, bioinformatics

Biomarker identification is one of the essential steps in omics data analysis for biomedical research. Significant advances have been achieved in the field of omics data and the ever-lower price have made it more and more easy to investigate the molecular features of multi-types of diseases at various levels, like animal models, bulk tissues, and single cells. The abundant information contained in the omics data provides solid foundation for preclinical research and the development of new disease treatment strategies, including understanding disease mechanisms, finding molecular targets, and identifying biomarkers. Following the success of our Research Topic on the first volume on biomarker identification in clinical research based on omics data analysis, we make further remarkable progress of soliciting more than 50 impressive research articles in our Volume II in this Research Topic.

Eighteen papers aimed at detecting biomarkers from omics data generated from various tumors and diseases. Ning et al. showed that ANXA2 expression was significantly correlated with immune infiltration and might potentially serve as a prognostic biomarker for tumors. Specifically, by performing differential expression analysis, Ning et al. revealed that a high expression of ANXA2 was associated with reduced overall survival, disease-specific survival and progression-free interval in seven cancers. They also found that ANXA2 expression was related to immune cell infiltration and immune-related pathways in cancers, suggesting that it can function as a potential target for immunotherapy in pan-cancer. With the help of network pharmacology and molecular docking technology, Chen et al. studied the therapeutic mechanism of 4-octyl itaconate (4-OI), a cell-permeable derivative of itaconate, and found that 4-OI treats sepsis by regulating hub genes. The enrichment analysis further revealed that 4-OI participated in inflammatory imbalance, immunosuppression, and oxidative stress in developing sepsis. In another study, Cui et al. characterized the role of serine racemase (SRR) as a prognostic biomarker in endometrial cancer. By conducting differential expression analysis and GEO data mining, Yu et al. identified the correlation between immune cell types and recurrent implantation failure (RIF). They also provided new immune-related hub genes that serve as potential targets for both diagnosis and treatment of RIF. Hu et al. characterized the function of olfactomedin-like 2B (OLFML2B), an olfactomedin domain-containing protein, by applying some bioinformatics pipelines, qPCR and immunohistochemistry. They discovered that OLFML2B had high expression in 14 cancers and there is a positive correlation with the prognosis of specific cancers. In addition, it also contributed to infiltrating various immune cells, like macrophages. This result uncovered that OLFML2B could be regarded as a biomarker for diverse tumors. In another study, Yao et al. utilized the single-cell RNA sequencing technology to screen cancer stem cells (CSCs) in order to develop better treatment strategies for muscle-invasive bladder cancer (MIBC). Based on GEO data sets, weighted gene coexpression network analysis (WGCNA) and pseudotime analysis revealed that DBI is the key gene in treating CSCs and acetaminophen can be used as a candidate drug targeting CSCs. Wen et al. proposed GAPDH as the most reliable reference gene for pan-cancer diagnosis in platelets by performing bioinformatics and functional analysis from the RNA-seq of platelets data set. Results of RT-qPCR and internal stability analysis software programs evidenced that GAPDH was the most stably expressed gene with high expression among all 95 candidate genes identified from RNA-sequence data. Ullah et al. revealed that ATPase (PSMC) family of genes had great potential in lung adenocarcinoma (LUAD) diagnosis and therapeutic measure developing. They found that multiple somatic mutations along the PSMC coding regions in LUAD tissues helped to screen potential patients. The correlation between the PSMC overexpression and LUAD patients’ poor overall and relapse-free survival (*p* < 0.05; HR: >1.3) and individual cancer stages (*p* < 0.001) further proved that PSMC was an ideal target for diagnosing LUAD. To investigate the role of the hepatitis A virus cellular receptor 2 (HAVCR2) gene in cancer immunity and prognosis, Li et al. investigated its expression patterns in pan-cancer and they discovered that the expression level of HAVCR2 has significant correlations with cancer immune infiltration, immune checkpoint genes, and immune marker genes. Results showed that T cell immunoglobulin mucin 3 (TIM-3), the expression of HAVCR2 may contribute to effects of immunotherapy. Cui et al. characterized functions of Anillin (ANLN), a unique scaffolding and actin-binding protein, in various cancers, like prognostic value. The expression level of ANLN was upregulated in most tumors and it particularly increased in early stages of 17 cancers. More importantly, ANLN is highly correlated with infiltration levels of most immune cells and it is a significant part of cell cycle, mitosis, cellular senescence and p53 signaling pathways, suggesting that it may become an important factor for pan-cancer diagnosis and treatment. To learn the detailed mechanism of fibrosis in the progress of heart failure (HF), Tao et al. filtered 1,187 fibrosis-related differentially expressed genes (DEGs) from the Gene Expression Omnibus (GEO) cohorts. Ten hub genes (PPARG, KRAS, JUN, IL10, TLR4, STAT3, CXCL8, CCL2, IL6, IL1β) were filtered by the protein-protein interaction (PPI) network and six of them had high diagnostic accuracy after receiver operating characteristic curve analysis. Quantitative real time PCR proved that these six selected genes can be used as biomarkers in heart failure (HF) diagnosis. Studies have described that DEAD-box helicase 5 (DDX5) gene played an important role in the modification of RNA structures. In order to specify its prognostic and immunological roles in pan-cancer, Liu et al. collected and analyzed data from six data sets, like The Cancer Genome Atlas (TCGA). They revealed that DDX5 closely correlated with its co-expressed genes in pan-cancers and it also associated with multiple cellular pathways, highlighting the importance of DDX5 in immunology and diagnosis of cancer. Based on a combined cohort, Wang et al. established an original immune signature called the IMS, which had correlation with significant immune activation, better prognosis, and increased immunotherapy responsiveness. They further discovered that the tumor microenvironment which contained higher IMS might have enriched with pathways related to glycolysis/gluconeogenesis, oxidative phosphorylation, and citrate cycle (TCA cycle), suggesting its great potential in managing head and neck squamous cell carcinoma (HNSCC) patients. Ye et al. screened three biomarkers (SERPINB2, TFPI2, and SLC9B2) for Crohn’s disease (CD) diagnosis and anti- tumor necrosis factor (TNF) medication outcomes prediction by using bioinformatics analysis and machine learning. Based on these targets, they further constructed a Sial-score which could distinguish between patients who had a good response to anti-TNF and those who had not, indicating that the filtered biomarkers aided in the process of CD treatment. Huang et al. applied various methods, like STIMATE algorithm, to investigate the microenvironment and prognostic targets in BRAF mutated SKCM patients. In addition, they also summarized that the dysregulation of immune function and immune cells may lead to the bad outcomes of BRAF mutated patients. In order to learn the mechanism of adenomyosis, Liu et al. found four hub genes including STEAP1, TOMM20, GLT8D2, and NME5 from two microarray datasets and their potential in adenomyosis diagnosis had been proved by qRT-PCR. Additionally, based on the immune infiltration analysis, they found that T helper 17 cells, CD56dim natural killer cells, monocytes, and memory B cell may result in the occurrence of adenomyosis. Mi et al. performed network analysis and differential analysis of functional similarities between androgen receptor (AR) and the synaptic protein postsynaptic density 95 (PSD95) using protein–protein interaction data to evaluate the effect of androgens on synaptic plasticity. The authors discovered that CaMKII played a critical role in mediating the rapid effect of androgen which regulates the synaptic protein PSD95. Kang et al. evaluated functional similarity between AD and T2DM differentially expressed genes (DEGs) through Gene Ontology (GO) semantic similarity, protein-protein interaction, and biological pathways. The study highlighted the common pathways and pathogenic genes shared by Alzheimer’s disease (AD) and type 2 diabetes mellitus (T2DM). Additionally, SLC2A2 had the potential to be utilized as an early warning and monitoring marker for Alzheimer’s disease in patients with type 2 diabetes mellitus (T2DM).

In terms of method development, Wan and Wang proposed IterMegaBLAST, an obfuscation method based on sequence similarity, to fast and reliably protect personal genomic privacy. In terms of utility accuracy and time complexity, the benchmarking results demonstrated that IterMegaBLAST showed superior performance compared to existing state-of-the-art methods. Xu et al. developed a robust signal recognition particle (SRP)-related joint model of LASSO regression, SVM-RFE and artificial neural network which specifically focused on the diagnosis of systemic sclerosis-associated pulmonary hypertension (SSc-PH). The joint model provides a powerful tool to explore and investigate the potential roles of SRP in the underlying disease mechanisms of SSc-PH, thereby facilitating precision and personalized medicine in the context of SSc-PH. In the field of microbial research, Zhou et al. proposed the PhageTailFinder algorithm based on a two-state hidden Markov model (HMM) to predict the probability of tail-related proteins and identify the putative tail modules in phages that were previously uncharacterized, which would contribute to the utilization of bacteriophages for therapeutic applications. Gan et al. introduced DBSCAN-SWA, integrating density-based spatial clustering of applications with noise (DBSCAN) and a sliding window algorithm (SWA), to predict prophage regions with high-throughput mode in bacterial genomes. It demonstrated better detection speed and efficiency compared with existing tools, which effectively addressed the increasing demands in the context of the exponential growth of microbial genome sequences. In addition, Zheng et al. developed comprehensive Cancer genome Consensus Annotation System (CCAS) at multi-omics level. CCAS enable multidimensional data annotation and functional analysis for 395 subtypes of 10 categories of cancers.

Panels or models were constructed based on molecular targets filtered from omics data to assess the dynamic of illness. Chen et al. showed the prognostic role of glycolysis and further developed a glycolysis-related signature in pancreatic cancer by combining single-cell and bulk transcriptomic data. The signatures can be used to effectively assess the risk subtypes of patients with pancreatic cancer and offer personalized patient management. For esophageal cancer, Guo et al. constructed a prognostic model based on immune-related genes. The model successfully identified that the low-risk group with esophageal cancer had better overall survival than the high-risk group. In another study on hepatocellular carcinoma, Hu et al. used a panel of E2F target gene signature to accurately predict the prognosis of the disease. In addition, a necroptosis-related lncRNA model was built by Luo et al. to predict the prognosis and immune response of colon cancer. From the Gene Set Enrichment Analysis (GSEA) results, they have also shown that the necroptosis-related lncRNAs were involved in the pathogenesis and progression of colon cancer, including the alteration of immune cell activities. Li et al. structured a TF-miRNA-hub gene network from KEGG pathway and PPI network and found three key feedback loops (MYC-miR-34a-5p-LDHA, YY1-miR-155-5p-HIF1A, and RELA-miR-93-5p-HIF1A) which highly related to the ovarian endometriosis (OE) mechanism. The result may provide insights for endometriosis (EMs) therapy. After multiple analysis, Wu et al. built ceRNA network with miR-224-5p, miR-30a-5p, and miR-204-5p at the center. It revealed that aniridia-associated keratopathy (AAk) is associated with immune cell infiltration. With machine learning methods, Miao et al. developed a prediction tool for prognosis and immunotherapy response prediction for LGG patients by capturing CNRG-based signature. Zhang et al. proposed the NEsubtype-panel based on the orderings of relative gene pairs expression to distinguish the histological subtypes for each NE sample. Composed of three signatures which can achieve high average concordance rate, the panel successfully help the complement of lung cancer diagnosis. By using WGCNA to link gene expression, hypoxia and angiogenesis, Liu et al. constructed a novel six-gene prognostic model for cervical cancer. The Kaplan-Meier analysis and ROC curves indicated the high predictive power of the tool. Song et al. tested their risk model for skin cutaneous melanoma through Necroptosis-related genes with almost the same methods and secure the usage in precise treatment. Through Lasso-Cox regression, Zhao et al. also built a model from six epithelial–mesenchymal transition-related genes for idiopathic pulmonary fibrosis biomarker identification while Liu and Liu further explored the role of hypoxia in diffuse large B cell lymphoma (DLBCL) hypoxia-related subtypes and signatures discovery for better treatment. Li et al. increase the accuracy in hepatocellular carcinoma diagnosis with a nucleotide metabolism-related prognostic model. Jiang et al. retrained age, pathologic stage and prognostic risk model and identified an applicable prognosis model for rectal adenocarcinoma (READ) with nine immune-related genes. Moreover, the age and mRNA factors are highly valued. Xia et al. used K-means clustering to divide copper ionophore–induced death (CID) subtypes when employing ESTIMATE and CIBERSORT algorithms to illustrate microenvironment of clear cell renal cell carcinoma (ccRCC) for cancer prediction model development. Zhou et al. instead, proved the significance of basement membrane-related genes and constructed a risk model of eight BMRGs, including COL4A4, FREM1, CSPG4, COL4A5, ITGB6, ADAMTS14, MMP17, and THBS4 for this cancer. For gastric cancer, as Wang et al. discovered from integrative analysis, TNFα-derived gene signature containing AKR1B1, CPVL, and CTSL might assist in its therapy. For colorectal cancer (CRC) detection, a eleven-gene signature related to lipid metabolism invented by Huang et al. will be useful.

Bioinformatics analysis has also facilitated the discovery of new mechanisms in clinical research. Ouyang et al. used bioinformatics methods to discover core RNA targets and competitive endogenous RNA (ceRNA) networks in Keratoconus. They predicted four core miRNAs and proposed a ceRNA network, which may highlight potential post-translational regulatory mechanisms of Keratoconus. Regarding thoracic aortic aneurysms and dissections (TAAD), Guo et al. performed whole-exome sequencing to identify its genetic origin in three Chinese families. They identified candidate genes including COL3A1, ACTA2, etc., which provided potential insights into the underlying disease mechanism. Through the use of integrated bioinformatics analysis and machine learning, Hong et al. constructed a ceRNA network mediated by MIR600HG/hsa-mir-21-5p, which participated in tuberculosis (TB) activation. Hong et al. also applied an elastic net regression model on the gene biomarkers to classify between active TB and latent ones. The validation results demonstrated promising generalizability across various host cases. Liu et al. proved that cuproptosis was critical to the progression of bladder cancer (BLCA) by building a cuproptosis-related (CR) score signature. They also showed that the CR score can predict the prognosis and evaluate therapeutic effects of BLCA. To investigate the mutational signatures of hepatocellular carcinoma (HCC), Wu et al. analyzed the affected coding and non-coding RNAs, and their regulatory network using genomic and transcriptomic data from The Cancer Genome Atlas (TCGA). The study identified key genes and pathways for mutational signature-specific HCC and some of the RNAs also act as prognostic markers to predict the survival outcome of HCCs with specific mutational signatures. For esophageal squamous cell cancer (ESCC) research, Li et al. employed propensity score matching (PSM) to eliminate the presence of selection bias between genders with ESCC. The study suggested that female patients with high total cholesterol (TC) level were found to have significant poor overall survival in stages III and IV and total cholesterol (TC) may served as an independent prognostic factor specifically in females. Zhang et al. identified three ESCC subtypes with specific immune profiles based on the expression patterns of ferroptosis-related genes (FRGs). Furthermore, the researchers defined a gene signature that could effectively characterize ferroptosis patterns and have potentials for predicting the response to immunotherapy. By leveraging the transcriptomic data, Chen et al. identified immune-related differentially expressed genes (DEGs) related to disease progression of burns and blunt trauma for subsequent analyses, including gene enrichment analysis, network analysis and clinical correlation analysis. These functional analyses highlighted the critical signaling pathways and core DEGs significantly associated with simultaneous dysregulation of immune cells in burns and blunt trauma diseases. Huang et al. investigated the common differentially expressed genes (DEGs) of infantile hemangiomas (IH) and venous malformations (VM) using microarray datasets. Functional analysis revealed the critical role of hub genes and signaling pathways in IH and VM, which could be biomarkers associated with the diagnosis and pathophysiology of vascular abnormalities. By leveraging the power of machine learning, Heng et al. identified three hub genes (PLIN, PPAP2A, and TYROBP) between rheumatoid arthritis (RA) and pigmented villonodular synovitis (PVNS) using least absolute shrinkage and selection operator (LASSO) logistic regression and random forest (RF). The hub genes demonstrated good diagnostic efficiency and significant association with immune infiltrating cells.

Identification of reliable biomarkers is the key step for personalizing medicine and therapy. Omics data generated from genomics, transcriptomics, proteomics, epigenomics, metagenomics, and metabolomics contribute to screening stable targets with high accuracy in prognosis and diagnosis of multi-diseases. With the advent of single cell technologies, some omics data analysis methods (Wan et al., 2020; Wang et al., 2022; Wang and Wan, 2023) have also been proposed to facilitate the biomarker identification in the single-cell basic, translational, and clinical research. In conclusions, we are deeply grateful for all the authors who have contributed to this Research Topic, and more importantly, who have enhanced our understanding of biomarker identification in various clinical research environments. We believe that with the development of bioinformatics omics data analysis and machine learning approaches, more exciting discoveries based on biomarker identification in clinical research will emerge in the near future to facilitate intelligent healthcare and precision medicine.
